# Study design and characteristics of the Luxembourg European Health Examination Survey (EHES-LUX)

**DOI:** 10.1186/s12889-018-6087-0

**Published:** 2018-10-11

**Authors:** Valéry Bocquet, Jessica Barré, Sophie Couffignal, Marylène d’Incau, Charles Delagardelle, Georges Michel, Marc Schlesser, Saverio Stranges, Andrea Kuemmerle, Maria Ruiz-Castell

**Affiliations:** 1 Competence Centre for Methodology and Statistics, Department of Population Health, Luxembourg Institute of Health, 1ab rue Thomas Edison, L-1445 Strassen, Luxembourg; 2 Epidemiology and Public Health Research Unit, Department of Population Health, Luxembourg Institute of Health, Strassen, Luxembourg; 3 Clinical and Epidemiological Investigation Center, Department of Population Health, Luxembourg Institute of Health, Strassen, Luxembourg; 4 Service de Cardiologie, Centre Hospitalier du Luxembourg, Luxembourg, Luxembourg; 5 Société luxembourgeoise de Diabétologie, Luxembourg, Luxembourg; 6 Service de Pneumologie, Centre Hospitalier du Luxembourg, Luxembourg, Luxembourg; 7 Department of Epidemiology & Biostatistics, Schulich School of Medicine & Dentistry, Western University, London, ON Canada; 8 Department of Family Medicine, Schulich School of Medicine & Dentistry, Western University, London, ON Canada

**Keywords:** European Health Examination Survey, Health outcomes, Luxembourg, Recruitment, Representativeness, Study design

## Abstract

**Background:**

The European Health Examination Survey in Luxembourg (EHES-LUX) is a population based survey performed from 2013 to 2015 with the aim to establish baseline information on the general health status of the Luxembourg population aged 25 to 64 years. The paper introduces the study design, recruitment method and representativeness of the sample, and summarizes the sociodemographic characteristics of participants and the prevalence of major health outcomes.

**Methods:**

EHES-LUX is based on a random sample representative of the population of Luxembourg based on gender, age and district of residence. The sample size of the study was determined to provide accurate prevalence estimates for major chronic conditions. During two visits, data were collected from participants through a questionnaire (personal data, health status and health care), medical examinations (anthropometric measures, electrocardiogram and spirometry) and biological analysis (blood, urine and hair). Means and frequencies were used to describe the general characteristics of the population and a one-way ANOVA to test the representativeness of the sample and the comparability of participants and non-participants.

**Results:**

A total of 1529 individuals participated in this study (participation rate of 24.1%). Differences between participants and non-participants based on gender, age and district of residence were corrected by sampling weights. The mean age (±SD) of participants was 44.9 (±10.1) years, of which 52.8% were women. Based on clinical measurements, nearly 20% of participants were obese and more than one in three participants were overweight. From measurements (respectively from self-report), 22.0% (respectively 12.2%) were hypertensive, 49.3% (respectively 22.5%) had hypercholesterolemia, 3.5% (respectively 3.7%) had diabetes and 7.6% (respectively 6.0%) had depressive symptoms.

**Conclusions:**

This nationwide epidemiological study on the general health status of Luxembourg residents provides updated prevalence estimates on a range of major health conditions. This information can be used by health authorities to evaluate policies and public health initiatives. At European level, prevalence data obtained by this study following the EHES-Reference Committee (EHES-RC) recommendations, will be comparable between European countries participating in this program.

**Electronic supplementary material:**

The online version of this article (10.1186/s12889-018-6087-0) contains supplementary material, which is available to authorized users.

## Background

Registries and population-based surveys are valuable sources of data on the health of populations, informing on a number of challenges in health systems, such as the prevalence of health outcomes and risk factors. Health Interview Surveys (HISs) and Health Examination Surveys (HESs) are currently the two main types of surveys. Information from HISs is obtained through interviews or self-administered questionnaires, whereas HESs also include objective measures such as clinical examination and/or biological samples [1, 2]. After the first HESs carried out in the 1950s and 1960s, many countries launched their own national health surveys [1, 3]. However, the lack of a consistent methodology greatly limited the comparison of results between countries. A sustainable health monitoring system was suggested by the Community Public Health Programme 2003–2008 of the European Union [4]. This resulted in the development of the European Health Survey System, which includes the European Health Interview Survey (EHIS) and the European Health Examination Survey (EHES) [5]. Hence, EHES, as a standardised representative HES of the adult population of European countries, has focused mainly on major chronic diseases and their risk factors as its core measures.

In Luxembourg, no HES of the general health status had been done, with the exception of ORISCAV-LUX, a national survey which focused on determining the prevalence of major cardiovascular diseases and their risk factors [6]. In 2013–2015 period, the Grand Duchy of Luxembourg performed an EHES (EHES-LUX) with the aim of obtaining validated health information along with the identification of the population’s health care needs. EHES-LUX is designed to produce national health indicators of significant clinical and public health relevance, including cardiometabolic conditions, mental health, respiratory parameters, women’s health, thyroid disorders, and exposure to pollutants.

The Grand Duchy of Luxembourg (afterwards named Luxembourg) is a small multicultural country located in the heart of Europe (bordered by Belgium, France and Germany). Foreigners make up for nearly half of the population, counting some 150 nationalities including those from the neighbouring countries. The country is divided into three geographic districts (Diekirch, Grevenmacher and Luxembourg) [7, 8].

This paper introduces the study design, recruitment process and representativeness of the EHES-LUX sample, and summarizes the sociodemographic profile of participants and the prevalence of major health outcomes in Luxembourg.

## Methods

### Study design

EHES-LUX is a population-based survey performed from 2013 to 2015 that aims to establish baseline information on the general health status of the Luxembourg population aged 25 to 64 years (as the minimum selected age range defined in EHES recommendations [9]). Individuals who met the inclusion criteria filled in a questionnaire. Physical measurements and biological samples were also performed.

### Inclusion criteria

Four inclusion criteria were applied in this study: 1) be included in the national health insurance registry, 2) be resident in Luxembourg, 3) be 25–64 years old, and 4) sign an informed consent form. People living in institutions (e.g. hospital, nursing home, prison) were not included in this study.

### Sampling design and calculation

Given the small size of the country, a one-stage sampling procedure was performed. In order to obtain a representative sample of the population, a random sample of residents of Luxembourg was defined stratified by age (25–34, 35–44, 45–54, 55–64 years old), gender (male and female) and district of residence (Luxembourg, Diekirch and Grevenmacher). The representative random sample was drawn from the national health insurance registry by the General Inspectorate of Social Security (IGSS). Despite its relevance in the stratification, nationality or country of birth was not provided by IGSS. With a 95% social coverage, this registry is considered the most complete list of inhabitants available in Luxembourg. The 5% remaining people are identified as being mainly the employees of the European Commission (around 12,000) who have their own health insurance system and also asylum seekers (around 1000 new individuals each year) who just arrived and among which almost half are on the way to be registered.

To reach the adequate power and precision to perform age-gender subgroup comparisons, and to observe a 5% prevalence of a specific condition in that subpopulation [1], a minimum of 130 participants were fixed for each age-gender subgroup. Based on this information and on the last Luxembourg census data in 2011 [10], the total required sample size was 1490 participants. Since the minimal participation rate in previous EHES studies was 25% [2] and given the general declining trend in participation rates over time in comparable surveys [11], the participation rate in this present study was assumed to be 23% corresponding to an estimation of the total number of participants to be invited of 6475. Additional file 1: Table S1 illustrates the projection of sample size calculation of each age-sex domain based on the 2011 census.

### Recruitment

A letter signed by the Ministry of Health and the Luxembourg Institute of Health (LIH), which realised the study, was sent to the randomly selected individuals in order to inform and invite them to participate in the survey. Along with the letter, an information booklet on the survey, a response card and a pre-paid envelope were also sent. Individuals who were interested in participating in the study were asked to send back the reply coupon enclosed. If no response was received after 3 weeks, non-respondents were contacted again by post. Individuals did not receive any financial incentive for participating in the study.

Those who should be agreed to participate were contacted to fix an appointment for the examination and the collection of biological samples. To facilitate the participants’ access to the study, three different survey sites were available in the country: the Luxembourg Institute of Health (in the middle of the country), the Centre Pontalize (in the North) and the Centre Hospitalier Emile Mayrisch (in the South). We decided to plan two visits despite the possible inconveniences (loss of follow-up), because the completion of the questionnaire and the examination was too long.

#### Study visit 1

At the examination centre, visit 1 included the completion of a self-administered questionnaire, a medical examination, and the collection of a hair sample. On average, the visit had a duration of approximately 2 h.

The questionnaire included information on the participant’s socioeconomic characteristics, health status (e.g. general health status, diseases and chronic conditions, accidents and injuries, absence from work (due to health problems), physical and sensory functional limitations, personal care activities, household activities, pain, mental well-being and sleep disorders), health care (e.g. use of inpatient and day care, use of ambulatory and home care, medicine use, preventive services, unmet needs for health care), health determinants (e.g. self-reported height and weight, physical activities / exercise, consumption of fruit and vegetables, social support, provision of informal care or assistance, tobacco use, alcohol consumption and drugs) and women’s health.

Interviews and medical examinations were conducted by trained clinical research nurses in English, German, French or Portuguese. Questionnaires were language-validated [12]. The validation process consisted in three steps: 1) the English version was translated to German, French or Portuguese by a certified translator whose the mother tongue was the target language; 2) the first version was back-translated to English by an English native speaker and compared to the original version; and 3) based on the discrepancies between the back-translated and the original versions, an improved version in German, French and Portuguese was created.

Examinations included blood pressure and anthropometric measurements, spirometry, visual acuity, thyroid function and an electrocardiogram.

Hair samples from each participant were obtained and stored at the Human Biomonitoring Research Unit of the LIH in order to measure pollution and pesticide consumption indicators in future research.

#### Study visit 2

A second visit was held at the laboratory where blood and urine samples were taken and analysed from each participant. Blood samples were used to measure plasma glucose, glycated haemoglobin (HbA1c), triglycerides, total and HDL cholesterol (LDL cholesterol calculated), creatinine, and thyroid function (TSH, anti-TPO). Urine samples were obtained to measure ioduria, creatinuria and microalbuminuria.

Results of medical examinations from visit 1 and biological analyses from visit 2 were evaluated and validated by a clinical committee (composed of a cardiologist, a pulmonologist and a diabetologist) who verified the presence or absence of anomalies.

### Eligibility status

Individuals were classified as: 1) eligible, if she/he met the inclusion criteria, 2) not eligible if she/he did not met the inclusion criteria or 3) unresolved (e.g. if the invitation letter was returned due to change of address, contacts were not possible or not successful, no information was available to assess the eligibility status). An eligible person was classified as participant if she/he had at least one valid examination measurement (e.g. height and weight) in addition to the completed questionnaire. If the individual contacted agreed to participate, it was recorded as a positive answer. If the eligible person refused to participate she/he was classified as non-participant.

### Definition of variables

From participant’s height (in meters) and weight (in kilograms), Body mass index (BMI) was calculated as the ratio of weight divided by the square of height. Self-reported and measured BMI were recorded. Subjective BMI was calculated based on participant’s self-reported value and measured BMI was calculated from the value measured by clinical research nurses. We categorised BMI as normal weight (< 25 kg/m^2^), overweight (25 to < 30 kg/m^2^) and obese (≥30 kg/m^2^).

Participants’ blood pressure was measured 3 times on the right arm in a sitting position [13]. Blood pressure was measured after a 5-min rest and with 1-min intervals between the following two measurements. Both systolic and diastolic pressure was calculated as the mean of his/her second and third measurements. Measured hypertension was defined as systolic/diastolic blood pressure greater than or equal to 140/90 mmHg. Self-reported hypertension was defined as hypertension being diagnosed by a physician. Information on medications for high blood pressure was obtained from the questions “*In the past 2 weeks, have you used any medicines that were prescribed to you by a doctor? Were these medications for high blood pressure?*”

Measured diabetes was defined as fasting plasma glucose higher than or equal to 126 mg/dL. Self-reported diabetes was defined as being diagnosed as diabetic by a physician. Information on glucose lowering medications was obtained from the questions “*In the past 2 weeks, have you used any medicines that were prescribed to you by a doctor? Were these medications for diabetes?*”

Hypercholesterolemia was defined as total cholesterol values higher than or equal to 200 mg/dL. Self-reported hypercholesterolemia was defined as being diagnosed by a physician as having high cholesterol. Information on blood cholesterol lowering medications was obtained from the questions “*In the past 2 weeks, have you used any medicines that were prescribed to you by a doctor? Were these medications for lowering blood cholesterol levels?*”

Depression was evaluated with the Patient Health Questionnaire (PHQ-9) defined by Kroenke et al. [14], and completed by participants. We defined depressive symptoms as a PHQ-9 score of greater than or equal to 10 (the maximum score is 27). Self-reported depression was defined as being diagnosed by a physician as having depression. Information on anti-depressive medications was obtained from the questions “*In the past 2 weeks, have you used any medicines that were prescribed to you by a doctor? Were these medications for depression?*”

### Privacy of participants

The study participants’ data privacy (confidentiality, integrity and availability) was maintained throughout the entire study. Each participant was assigned a participant number. Identifiable paper or electronic source documents (e.g. signed informed consent forms, medical feedback letter and appointment books) were kept in a secure location under the responsibility of the medical investigator. We used Ennov Clinical® as the data management system and defined data access rights to each survey member (data entry clerk, data manager, statistician, project manager, etc) according to their responsibilities. Moreover, any data entry / modification was automatically logged to a non-changeable audit trail.

### Statistical analysis

For each age and gender stratum, sampling weights were used to account for differences between participants and non-participants (See details in Additional file 2: Table S2). Moreover, as Luxembourg is a multicultural country, a comparison between participants and Luxembourg population according to the country of birth was performed. Weights were calculated from the selection probabilities (with 2011 Luxembourg census data as a reference) and were adjusted for non-response.

Response rates were defined as the ratio of the number of positive answers over the number of invitations sent. Participation rates were defined as the ratio of the number of participants over the number of eligible and unresolved individuals [15].

The representativeness of the sample and the comparability of participants and non-participants were analysed with a one-way ANOVA test. Gender and district effects on the number of study participants were estimated by a Poisson regression analysis.

Stratification and all statistical analyses were carried out using SAS version 9.4 (SAS Institute Inc., Cary, NC, USA). A two-sided *P* < 0.05 was considered statistically significant.

## Results

### Recruitment process

The recruitment process took place from 1st February 2013 to 15th January 2015.

**Fig. 1:**
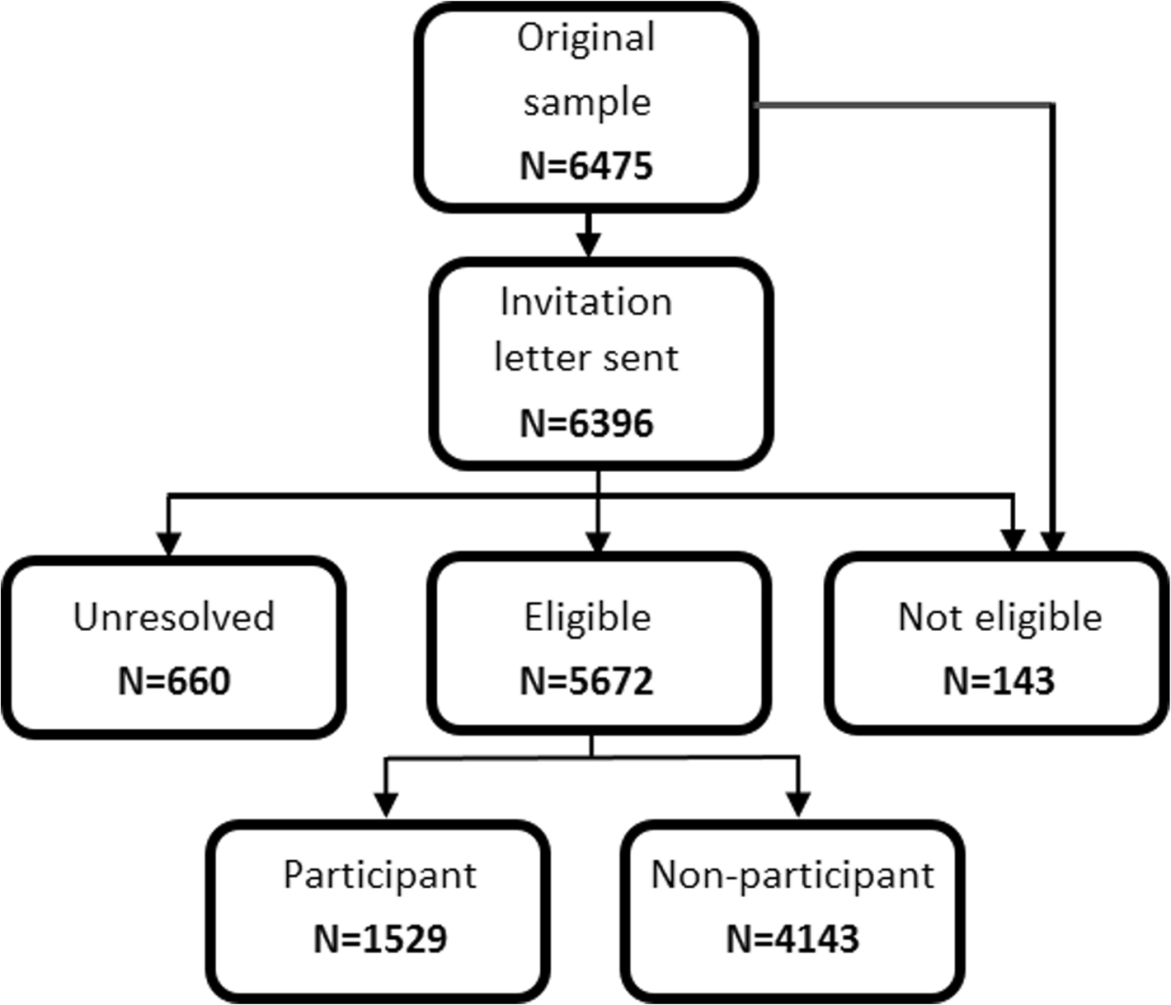
Flow chart of the recruitment process

A total of 6475 individuals were drawn and 6396 invited after a first step of exclusions for age (older than 64 at the moment of the visit 1) or invalid address (Fig. 1). From these, 5672 (87.6%) were eligible, 143 (2.2%) were not eligible (e.g. exclusions) and 660 (10.7%) unresolved (e.g. invalid addresses, lost follow-up or other reasons). Of the 5672 eligible individuals, 1529 individuals participated in the study and 4143 did not participate (e.g. negative or no answers).

A description of response rate and participation rate is shown in Table 1. There was an 18% response rate for first invitations. First and second reminders increased the response rate up to nearly 30%. Out of the 1902 total positive answers, 1529 individuals participated in the study, corresponding to an overall participation rate of 24.1% (men: 22.6%; women: 25.7%), which was in line with expectations.

**Table 1:** Impact of response measures

	N	Response rate
		*N* = 6396
First invitation	1180	18.4%
First reminder	514	8.0%
Second reminder	208	3.3%
Total positive answers	1902	29.7%

### Response rate by gender and by district of residence

Response rate (Table 2) varied significantly according to gender (*p* = 0.01) and district of residence (*p* < 0.0001). Independent of the district of residence, the response rate was systematically lower for men than for women. The highest rates were observed in the region of Grevenmacher, in both men and women.

**Table 2:** Response rate by gender and by district of residence (*N* = 1902)

District	Positive answer	
	Male	Female
Diekirch	122 (25.2%)	149 (33.1%)
Grevenmacher	121 (32.6%)	131 (35.5%)
Luxembourg	649 (27.3%)	730 (31.1%)
Total	892 (27.6%)	1010 (31.9%)

### Sample representativeness

Table 3 shows the comparison between participants and Luxembourg population according to age, gender, district and country of birth. There was an association between gender and participation (*p* < 0.01) with an over-representation of female participants compared to Luxembourg population. There was an association between age and participation (*p* < 0.01) with an over-representation of 45–54 participants and an under-representation of 25–34 participants compared to Luxembourg population. There was an association between the district of residence and participation (*p* = 0.02) with an over-representation of participants from the East of Luxembourg (Grevenmacher) and an under-representation of the North of Luxembourg (Diekirch) compared to Luxembourg population. There was an association between the country of birth and participation (*p* < 0.01) with an over-representation of participants born in Luxembourg and an under-representation of participants born in Portugal.

**Table 3:** Relative percentage of respondents in each district by gender, age and district of residence

	Participants	2011 Census	Non-participants	P*	P**
	*N* = 1529	*N* = 290,221	*N* = 4803		
Gender				< 0.01	< 0.01
Male	722 (47.2%)	143,101 (49.3%)	2473 (51.5%)		
Female	807 (52.8%)	147,120 (50.7%)	2330 (48.5%)		
Age				< 0.01	< 0.01
25–34	314 (20.5%)	73,520 (25.3%)	1446 (30.1%)		
35–44	461 (30.2%)	82,094 (28.3%)	1488 (31.0%)		
45–54	461 (30.2%)	78,122 (26.9%)	1115 (23.2%)		
55–64	293 (19.2%)	56,485 (19.5%)	754 (15.7%)		
District				0.02	< 0.01
Diekirch	208 (13.6%)	42,662 (14.7%)	711 (14.8%)		
Grevenmacher	214 (14.0%)	34,322 (11.8%)	518 (10.8%)		
Luxembourg	1107 (72.4%)	213,237 (73.5%)	3574 (74.4%)		
Country of birth				< 0.01	NA
Luxembourg	799 (52.3%)	139,201 (48.0%)	NA		
Portugal	222 (14.5%)	47,194 (16.3%)	NA		
France	110 (7.2%)	20,796 (7.2%)	NA		
Other countries	398 (26.0%)	83,030 (28.6%)	NA		

P* is related to the distribution difference between participants and 2011 Census;

P** is related to the distribution difference between participants and non-participants

*NA* not available

### Comparison between participants and non-participants

Table 3 shows also the comparison between participants and non-participants according to age, gender and district. There was an over-representation of female participants (*p* < 0.01), individuals aged 45–54 (age effect, *p* < 0.01) and individuals from the East of Luxembourg (Grevenmacher) (district effect, *p* < 0.01) and an under-representation of 25–34 participants and of the North of Luxembourg (Diekirch).

### General characteristics of participants

Among the 1529 individuals participating to this study, 1469 came to the second visit. Additional file 3: Table S3 shows the socio-demographic characteristics of participants attending to only visit 1 and attending to both visits. We observed small differences on age and job status between both samples. About half of the 1529 participants were women (52.8%). The mean age was 44.9 (±10.1). Nearly three in four participants resided in the district of Luxembourg and were working. Participants were mainly married or in a civil union (66.1%). Moreover, one fourth of participants were not working (compared to 28.7% not working in 2014 in Luxembourg population aged 20–64 [10]).

Health status of participants after excluding pregnant women (*N* = 21) are represented in Table 4. We observed differences between results from self-reporting and study measurements. Nearly half (48.5%) of participants had a normal weight based on self-reported values whereas the rate from the measured value was 42.8%. One in every eight participants (12.2%) reported having hypertension compared with 22.0%, defined from measurements. High cholesterol values ranged from 22.5% (self-reporting) to nearly 50% (measured).

**Table 4:** Health status from three modes of detection (*N* = 1508)

		Self-reporting	Study measurement	Reported medications
BMI	Normal weight (< 25 kg/m2)	731 (48.5%)	645 (42.8%)	N/A
	Overweight (25-30 kg/m2)	521 (34.5%)	555 (36.9%)	N/A
	Obese (≥30 kg/m2)	256 (17.0%)	306 (20.3%)	N/A
Hypertension		184 (12.2%)	331 (22.0%)	150 (10.0%)
High cholesterol		339 (22.5%)	744 (49.3%)	205 (13.6%)
Diabetes		56 (3.7%)	53 (3.5%)	53 (3.5%)
Depressive symptoms		115 (7.6%)	91 (6.0%)	79 (5.2%)

Data expressed as N (%). N/A, not applicable

The number of participants with diabetes defined with blood glucose levels reporting taking medications for diabetes were slightly lower (*N* = 53) than those who reported having being diagnosed by a physician (*N* = 56). Depressive symptoms were reported by 7.6% of participants based on self-reporting and by 6.0% based on the PHQ-9 questionnaire.

## Discussion

For the first time in Luxembourg, it was possible to carry out a population based study with standardised measures common to the rest of European Member States. EHES provides a general overview of the health status of the population.

The sample was not representative of the population residents of Luxembourg in terms of age, gender and district, since there were a few significant differences between our participants and the general country population. However, these small dissimilarities were corrected by using a weight on each stratum to avoid biased population estimates. As Luxembourg is a multicultural country, it would be relevant to stratify on ethnicity but this information was not available for non-participants. The comparison between our study and the general population of Luxembourg showed slight but significant differences according to the country of birth, with more native participants in the EHES sample. As already observed in some studies [16, 17], ethnic minorities are often mentioned as under-represented in medical research.

Unlike EHIS, EHES-LUX includes several additional health modules, and objective measurements such as clinical examination and biological samples. Our results showed that self-reported and measured information were complementary: the prevalence of hypertension includes those who reported having being diagnosed as hypertensive by a doctor (self-reported), along with those who at the time of the examination had high blood pressure. As a high percentage of the population are not aware of being hypertensive [13], information on self-reported hypertension underestimates the real situation. On the other hand, medications for high blood pressure would hide real cases of hypertension that can be detected using a self-reported information. Both sources of information shows that the number of participants with a specific disease differed according to the methodology used. A well-known and studied example is the case of self-reported weight and height, which are usually underestimated and overestimated respectively [18–20]. The same is observed for blood pressure, hypertension or cholesterol [21].

EHES-LUX provides national validated health information along with the identification of the population health care needs. No HES of general health status had taken place in Luxembourg, with the exception of ORISCAV-LUX [6], a national survey which focused on determining the prevalence of major cardiovascular diseases and their risk factors conducted in 2007/2009 - nearly 10 years prior to the current study. Information from EHES-LUX will help to develop national and European health indicators, identify the most prevalent diseases and at-risk behaviours, evaluate health behaviours and evaluate the impact of implemented health programmes.

A limitation of EHES-LUX was the low participation rate without the possibility to know the reasons for not participating. Galea et al. [22] showed that the main issue relating to study non-participation is the potential for non-participation bias. It is the case when the extent of non-participation is associated with the exposure or the outcome. The importance of bias is based on the difference between participants and non-participants [23]. In the present study, it was not possible to measure a possible bias on non-respondents’ health with the exception of administrative variables such as age, gender and district of residence. Fortunately, a low participation rate is not always associated with a selection bias [24]. The participation rate of EHES-LUX was comparable with other EHES studies for which the value ranged from 16 to 57% for men, and from 31 to 74% for women [25, 26]. Moreover, our participation rate is not remarkably different than that reported by previous observational studies in Luxembourg [6]. Alkerwi et al. noticed that this value order is realistic for this type of nationwide population-based survey with numerous sections covering a wide aspect of participants’ healthcare. As observed by Morton et al. [11] in a retrospective review of 355 articles in epidemiology, there is a decline in participation rates over time. Other studies [22, 27] noticed also that participation rates have declined steeply in recent years. Morton et al. [27] stated that a low participation rate does not necessarily translate in a lack of quality and/or validity of a study. Beyond participation rate, describing methods of recruitment and degree of representativeness of participants compared with non-participants are requisite to confirm quality and/or validity of a study [27]. As it was not possible in this current study to investigate the reasons for not participating, we can only speculate on potential drivers for a low participation rate. For example, there could be psychological factors with individuals who fear needles or medical exams [28], or who refuse to know from which disease they may suffer [29]. Other reasons include the lack of timely feedback on study findings to participants due to the reluctance of researchers to reveal relevant information before an international publication. As a consequence, the dissemination of the main results in press can wait for numerous months. Studies have shown that individuals could also consider that results do not benefit them [29] or that they are not interested in research or science in general [29–31]. Among other reasons, there is the possibility that research studies were too far away from their daily concerns [29] preferring to receive individual conclusions instead of general information on the population. In some cases, it could also be related to too large broad-spectrum healthcare objective (as in EHES-LUX) where the health objectives concern multiple diseases. Individuals may not feel an emotional interest in this type of study compared with a more focused aim such as cancer research [32]. Finally, due to Luxembourg population size, the request of participation in multiple surveys (socio-economic, public health, etc.), leads people to be less inclined to participate [33].

## Conclusion

EHES-LUX is the first population based study in Luxembourg with standardised measures common to the rest of European Member States. This study was an opportunity to provide updated national prevalence estimates on a range of major health conditions including cardio-metabolic outcomes, mental health, respiratory parameters, women’s health, thyroid disorders, exposure to pollutants and limitations to accessing health care services. It also highlighted the need to use complementary information on individuals’ health status to get accurate prevalence estimates for major chronic conditions: self-reported data, biological and anthropometric measurements and medications. Together with the information gathered from registries and other administrative sources, EHES-LUX results will provide evidence-based data to the European and Luxembourg health authorities. The results will be used at policy level to develop strategies and implement targeted interventions to improve health and health care services as well as to promote healthy behaviours among high-risk individuals and the population at large.

## Additional files

Additional file 1: **Table S1.** Projection of sample size calculation of each age-sex domain based on the 2011 census. The table shows the resident population of Grand-Duchy of Luxembourg based on the 2011 census, the invited individuals and the projection of participating individuals - broken down by gender, age and district. (DOCX 27 kb)

Additional file 2: **Table S2.** Weighting associated to each stratum (gender, age and district). The table shows weights used to account for differences between participants and non-participants. (DOCX 25 kb)

Additional file 3: **Table S3.** Socio-demographic characteristics of individuals attended only visit 1 and both visits. The table shows the socio-demographic differences between participants attending only visit one versus participants attending both visits. (DOCX 25 kb)

## Abbreviations

95%CI: 95% Confidence interval
BMI: Body mass index
CRF: Case report forms
EHES-LUX: European Health Examination Survey in Luxembourg
HbA1c: Glycated haemoglobin
HES: Health Examination Survey
HIS: Health Interview Survey
IGSS: General Inspectorate of Social Security
LIH: Luxembourg Institute of Health
SD: Standard deviation
TPO: Thyroid peroxidase antibody
TSH: Thyroid stimulating hormone
