# Supplementary material for: Study design and characteristics of the Luxembourg European Health Examination Survey (EHES-LUX)
Source: BMC Public Health. 2018 Oct 11;18:1169. doi: 10.1186/s12889-018-6087-0 (PMC6182799; doi:10.1186/s12889-018-6087-0)
Supplement: Supplementary file 1 — Table S1. Projection of sample size calculation of each age-sex domain based on the 2011 census. The table shows the resident population of Grand-Duchy of Luxembourg based on the 2011 census, the invited individuals and the projection of participating individuals - broken down by gender, age and district. (DOCX 27 kb) [file 12889_2018_6087_MOESM1_ESM.docx]

**Additional file 1: Table S1.** Projection of sample size calculation of each age-sex domain based on the 2011 census

| **Gender** | **Age** | **District** | | | | | **TOTAL** |
| --- | --- | --- | --- | --- | --- | --- | --- |
|  |  | **Luxembourg** | **Diekirch** | | **Grevenmacher** | |  |
| Resident population of Grand-Duchy of Luxembourg (census 2011) | | | | | | | |
| Male | 25-34 | 27967 | 4926 | | 3732 | | 36625 |
|  | 35-44 | 30616 | 6062 | | 4841 | | 41519 |
|  | 45-54 | 28642 | 6507 | | 5067 | | 40216 |
|  | 55-64 | 20799 | 4328 | | 3633 | | 28760 |
| Female | 25-34 | 28022 | 4946 | | 3927 | | 36895 |
|  | 35-44 | 29490 | 6065 | | 5020 | | 40575 |
|  | 45-54 | 27418 | 5829 | | 4659 | | 37906 |
|  | 55-64 | 20283 | 3999 | | 3443 | | 27725 |
| TOTAL | | | | | | | 290221 |
| Invited individuals | | | | | | | |
| Male | 25-34 | 679 | 135 | 96 | | 911 | |
|  | 35-44 | 718 | 159 | 124 | | 1001 | |
|  | 45-54 | 587 | 114 | 95 | | 796 | |
|  | 55-64 | 420 | 80 | 65 | | 565 | |
| Female | 25-34 | 672 | 135 | 104 | | 911 | |
|  | 35-44 | 706 | 144 | 118 | | 967 | |
|  | 45-54 | 560 | 104 | 89 | | 753 | |
|  | 55-64 | 430 | 78 | 64 | | 572 | |
| TOTAL |  |  |  |  | | 6475 | |
| Projection of participating individuals | | | | | | | |
| Male | 25-34 | 156 | 31 | 22 | | 209 | |
|  | 35-44 | 165 | 37 | 29 | | 231 | |
|  | 45-54 | 135 | 26 | 22 | | 183 | |
|  | 55-64 | 97 | 18 | 15 | | 130 | |
| Female | 25-34 | 155 | 31 | 24 | | 210 | |
|  | 35-44 | 162 | 33 | 27 | | 222 | |
|  | 45-54 | 129 | 24 | 20 | | 173 | |
|  | 55-64 | 99 | 18 | 15 | | 132 | |
| TOTAL |  |  |  |  | | 1490 | |
