# Supplementary material for: Study design and characteristics of the Luxembourg European Health Examination Survey (EHES-LUX)
Source: BMC Public Health. 2018 Oct 11;18:1169. doi: 10.1186/s12889-018-6087-0 (PMC6182799; doi:10.1186/s12889-018-6087-0)
Supplement: Supplementary file 2 — Table S2. Weighting associated to each stratum (gender, age and district). The table shows weights used to account for differences between participants and non-participants. (DOCX 25 kb) [file 12889_2018_6087_MOESM2_ESM.docx]

**Additional file 2: Table S2.** Weighting associated to each stratum (gender, age and district)

| **Gender** | **Age** | **District** | | |
| --- | --- | --- | --- | --- |
|  |  | **Luxembourg** | **Diekirch** | **Grevenmacher** |
| Male | 25-34 | 1.45882 | 1.23582 | 1.31078 |
|  | 35-44 | 0.99566 | 1.27748 | 0.72869 |
|  | 45-54 | 0.93725 | 1.26968 | 0.65110 |
|  | 55-64 | 1.06386 | 1.42510 | 1.27601 |
| Female | 25-34 | 1.15337 | 1.08573 | 0.82756 |
|  | 35-44 | 0.85837 | 0.99853 | 1.01721 |
|  | 45-54 | 0.95662 | 0.71417 | 0.64593 |
|  | 55-64 | 0.89049 | 1.05342 | 0.95469 |
