# Supplementary material for: Study design and characteristics of the Luxembourg European Health Examination Survey (EHES-LUX)
Source: BMC Public Health. 2018 Oct 11;18:1169. doi: 10.1186/s12889-018-6087-0 (PMC6182799; doi:10.1186/s12889-018-6087-0)
Supplement: Supplementary file 3 — Table S3. Socio-demographic characteristics of individuals attended only visit 1 and both visits. The table shows the socio-demographic differences between participants attending only visit one versus participants attending both visits. (DOCX 25 kb) [file 12889_2018_6087_MOESM3_ESM.docx]

**Additional file 3: Table S3.** Socio-demographic characteristics of individuals attended only visit 1 and both visits

|  |  | Only visit 1 (%)  N=1529 | Both visits (%)  N=1469 |
| --- | --- | --- | --- |
|  |  |  |  |
| Gender | Women | 807 (52.8%) | 776 (52.8%) |
|  | Men | 722 (47.2%) | 693 (47.2%) |
|  |  |  |  |
| Age | 25-34 | 314 (20.5%) | 297 (20.2%) |
|  | 35-44 | 461 (30.2%) | 444 (30.2%) |
|  | 45-54 | 461 (30.2%) | 443 (30.2%) |
|  | 55-64 | 293 (19.2%) | 285 (19.4%) |
|  |  |  |  |
| District | Luxembourg | 1107 (72.4%) | 1067 (72.6%) |
|  | Diekirch | 208 (13.6%) | 196 (13.4%) |
|  | Grevenmacher | 214 (14.0%) | 206 (14.0%) |
|  |  |  |  |
| Civil status | Never married or in a civil union | 307 (20.1%) | 298 (20.3%) |
|  | Married or in a civil union | 1009 (66.0%) | 971 (66.1%) |
|  | Widow | 28 (1.8%) | 25 (1.7%) |
|  | Divorced | 185 (12.1%) | 175 (11.9%) |
|  |  |  |  |
| Education | Primary | 377 (24.7%) | 365 (24.9%) |
|  | Secondary (finish) | 582 (38.2%) | 556 (38.0%) |
|  | Tertiary | 565 (37.1%) | 543 (37.1%) |
|  |  |  |  |
| Job status | Not working | 355 (23.2%) | 346 (23.6%) |
|  | Working | 1173 (76.8%) | 1122 (76.4%) |
|  |  |  |  |

Data expressed as N (%).
